# Feasibility and Acceptability of the Cancer-Specific PRONTO Protocol for Nutritional Risk Screening in Outpatient Oncology Cancer Care: A Pilot Study

**DOI:** 10.3390/cancers17223697

**Published:** 2025-11-19

**Authors:** Darío Sánchez-Cabrero, Jaime Rubio, Jorge Durá Esteve, Laura Guzmán-Gómez, Germán Guzmán-Rolo, Cristina Grande, Andrea Martín Aguilar, Pablo Pérez-Wert, Ana Pertejo, Suela Sulo, Amy R. Sharn, Samara Palma Milla, Carolina Dassen, Maurizio Muscaritoli

**Affiliations:** 1 Biomarkers and Experimental Therapeutics in Cancer, Instituto de Investigación Sanitaria del Hospital Universitario La Paz, 28046 Madrid, Spain; 2 Medical Oncology Department, La Paz University Hospital, 28046 Madrid, Spain; 3 Cancer Unit for Research on Novel Therapeutic Targets, Oncohealth Institute, IIS-Fundación Jiménez Díaz, UAM, 28040 Madrid, Spain; 4 Medical Oncology Department, University Hospital “Fundación Jiménez Díaz”, 28040 Madrid, Spain; 5 Radiation Oncology Department, University Hospital “Fundación Jiménez Díaz”, 28040 Madrid, Spain; 6 Global Medical Affairs and Research, Abbott Nutrition, 28050 Madrid, Spain; 7 Global Medical Affairs and Research, Abbott Nutrition, Chicago, IL 60064, USA; 8 Global Medical Affairs and Research, Abbott Nutrition, Columbus, OH 43219, USA; 9 Clinical Nutrition and Dietetics Unit, Endocrinology and Nutrition Department, Hospital Universitario La Paz, Biomedical Research Institute (IdiPAZ), 28049 Madrid, Spain; 10 Service of Endocrinology and Nutrition, University Hospital Fundación Jiménez Díaz, 28040 Madrid, Spain; 11 Department of Translational and Precision Medicine, Sapienza University of Rome, 00185 Rome, Italy

**Keywords:** cancer, nutritional risk, PRONTO, screening, sarcopenia, usability

## Abstract

**Background**: Malnutrition or its risk is common among patients with cancer (from 15% to 90%) and can negatively impact treatment outcomes and quality of life. Early detection and nutritional intervention are essential to improve clinical, health, and economic outcomes. This study evaluates the PRONTO (PROtocol for NuTritional risk in Oncology), a novel tool designed for the early identification of nutritional risk in patients with cancer. **Objectives**: To assess the feasibility of the PRONTO in detecting nutritional risk among newly diagnosed oncology patients. **Methods**: This cross-sectional, observational pilot study included 200 patients from two oncology centers in Madrid, Spain. The PRONTO was applied during the first oncology visit. Nutritional risk was assessed using PRONTO and nutritional status was evaluated with the Global Leadership Initiative on Malnutrition (GLIM) criteria. Tool acceptability was measured using the System Usability Scale (SUS) and Net Promoter Score (NPS) among participating oncologists. **Results**: The PRONTO identified 62.0% of patients at nutritional risk, while malnutrition was confirmed in 57.0% of patients using GLIM criteria. The PRONTO demonstrated high sensitivity (90.4%) and specificity (75.6%) compared to the GLIM. Oncologists rated the PRONTO highly, with a SUS score of 87.9 and an NPS of 9.1, indicating strong usability and recommendation potential. **Conclusions**: The PRONTO is a feasible and practical tool for early nutritional risk screening in oncology settings. Its simplicity and efficiency support its integration into routine clinical practice. The high prevalence of nutritional risk underscores the need for early screening to guide timely nutrition care. Further research with larger and diverse groups of patients and oncologists is needed to validate scalability and assess the impact of nutritional interventions on patient outcomes.

## Simple Summary

Malnutrition or its risk is common in people with cancer and can negatively affect their treatment and quality of life. However, it is often not detected early enough. This study tested a new screening tool called PRONTO (PROtocol for NuTritional risk in Oncology), designed to help oncologists quickly identify patients at risk of malnutrition during their first oncology visit. The tool is simple, fast, and easy to use, and it asks patients about recent weight loss, appetite, and strength. In this pilot study, the PRONTO successfully identified most patients who were later confirmed to have malnutrition using standard methods. Oncologists also found the tool highly usable and would recommend it to colleagues. These results suggest that the PRONTO could help improve early detection of nutritional problems in cancer care, allowing for timely support and better outcomes. Further research is needed to confirm its use in larger and more diverse patient groups and oncologist subspecialties.

## 1. Introduction

Malnutrition, regardless of the criteria used to define it, is independently associated with morbidity and mortality, particularly in patients with cancer [1,2,3]. In developed countries, the prevalence is estimated to be 15–40% at diagnosis and 80–90% in those with advanced disease [2,3]. At hospital discharge, more than a third of oncology patients are at risk for malnutrition [4]. Cancer type, cancer stage, primary tumor location, type and extension of metastases and administered antineoplastic treatment, and patient age are all factors that are related to the percentage of oncological patients who may suffer from malnutrition [5]. Cancer-related malnutrition differs from starvation-related malnutrition, as it results from a combination of anorexia, physical issues (e.g., mouth ulcers and pain), and metabolic dysregulation, which may be caused by the cancer itself or by its treatment(s) [6,7], all of which may make meeting nutrition needs difficult throughout the treatment journey.

Metabolic and nutritional abnormalities in patients with cancer are the drivers of cachexia. Cancer cachexia is a multifactorial syndrome marked by involuntary loss of muscle mass, often with fat loss, systemic inflammation, and negative protein–energy balance [8]. It ranges from pre-cachexia to refractory cachexia, leading to sarcopenia in 20–70% of patients depending on tumor type, characterized by muscle mass depletion, reduced physical function, and increased treatment toxicity [9]. Affecting 50–80% of patients, cachexia diminishes quality of life and prognosis, contributing to at least 20% of cancer deaths [10]. Consensus exists that cancer-related cachexia should be considered a type of disease-related malnutrition with inflammation [11,12].

Malnutrition has both prognostic and socioeconomic impacts, particularly in solid tumor patients. For this reason, the early detection of malnutrition for timely intervention can be critical in enhancing treatment tolerability, reducing the risk of treatment complications, and improving long-term treatment effectiveness, including survival, quality of life, and function [13]. Rapid and early detection of malnutrition in these patients for timely intervention can be critical in enhancing treatment tolerability, reducing the risk of postsurgical complications, and improving long-term treatment effectiveness: including survival, quality of life (QoL), and function [13].

The European Society for Clinical Nutrition and Metabolism (ESPEN) guidelines on nutrition in patients with cancer [14] have emphasized the importance of incorporating nutritional screening, evaluation, treatment, and continuous follow-up as an integral part of care. Currently, nutritional risk screenings are not a standardized approach for oncology physicians, and among European countries, screening rates are anywhere from 10 to 39% [15,16,17]. This limited implementation may be partly explained by a low perceived impact of nutrition on patient outcomes, time constraints, and insufficient training in nutritional management during oncology specialization. Establishing a standardized method for oncologists is essential to easily and promptly recognize patients beginning or undergoing antineoplastic therapy who may be at risk of malnutrition and should not replace current dietitian tools. Instead, a standardized method could provide a simple nutritional screening tool for optimizing oncology patient care [13].

To date, there are no universally accepted nutritional screening methods for oncology patients, although some have been validated in this population such as the Malnutrition Screening Tool (MST), the Malnutrition Universal Screening Tool (MUST), and the Nutritional Risk Screening (NRS-2002) [2]. The MUST has been used to evaluate the risk of malnutrition based on three independent criteria: acute disease effect, involuntary weight loss, and body mass index (BMI) [18]. The NRS-2002 is designed to identify patients who would benefit from nutritional support [19] and the MST includes questions regarding involuntary weight and appetite loss [20]. Depending on the scientific society or clinical pathway referenced, one method or the other has been suggested [2].

The PROtocol for NuTritional risk in Oncology (PRONTO) provides the opportunity to simply and rapidly identify oncological patients at nutritional risk or those with muscle loss earlier in the cancer journey, thereby facilitating their assessment and follow-up by the multidisciplinary team [13]. The PRONTO was developed by an expert panel of nutrition specialists and practicing oncologists to be used in various oncology settings, where indicated. The PRONTO is an evidence-based, oncology-specific awareness tool, designed for early identification of nutritional risk signs in patients with a cancer diagnosis. Therefore, its use is intended for the oncology setting and patients with cancer.

The PRONTO is a quick and efficient screening tool designed to enable healthcare professionals to gain an understanding of the patient’s nutritional risk status in under five minutes. It focuses on three domains including (1) body weight loss; (2) appetite and food intake; and (3) strength and mobility. These domains are assessed through three simple yes/no questions, providing a rapid snapshot of an oncological patient’s nutritional risk status.

• **Body Weight Loss**: Have you unintentionally lost weight (5% to 10% or more) in the last 3–6 months/since our last consultation?
• **Appetite and Food Intake**: Have you been eating less than usual in the last week/since our last consultation?
• **Strength and Mobility**: Have you lost strength, or do you feel weaker than usual/since our last consultation?

Note: Strength and mobility are intended as a proxy of muscle mass and function.

Screening using the PRONTO is considered positive for nutritional risk if one of the above questions has an affirmative response. Following an affirmative response, a detailed nutritional assessment is recommended to confirm the diagnosis of malnutrition [13].

The PRONTO can be implemented prior to initiating any antineoplastic therapy regardless of disease type or stage. Additionally, its simplicity and efficiency make it suitable for ongoing surveillance to detect emerging nutritional concerns throughout the treatment process. Early identification of patients at risk of malnutrition and/or muscle depletion will enable further evaluation using established malnutrition criteria such as the GLIM, or follow-up with dietitians or the multidisciplinary care team. Existing GLIM consensus criteria are precise, sensitive, and specific to the outpatient setting with oncology patients [9,10] and have also been validated for use in elderly patients with cancer not only to assess malnutrition but also to predict survival and other health outcomes [21,22].

The PRONTO does not seek to replace existing guidelines for comprehensive nutritional evaluation or clinical tools used to diagnose malnutrition but aims to validate and facilitate the recognition of nutritional risk in medically complex patients with cancer in daily medical oncology and radiotherapy practice. It aims to support oncologists in identifying patients at risk of malnutrition early and prevent nutritional decline through a straightforward, evidence-based, and cost-effective approach, without affecting the daily schedule of medical or radiation oncologists.

A relevant aspect for assessing the feasibility of the PRONTO in clinical practice, apart from its ability to adequately screen patients with nutritional risk, is the usability of the tool [23]. High usability values will result in greater implementation of the tool by the oncology community. Usability can be defined as the degree to which a product can be used by specific users to achieve effective and efficiently specific goals, while providing user satisfaction in a context of use [24]. We hypothesized that the PRONTO screening tool would demonstrate high usability among oncologists, reflected in positive System Usability Scale (SUS) and Net Promoter Score (NPS) ratings, supporting its feasibility for integration into routine oncological practice.

Thus, the present study seeks to analyze the feasibility and acceptability of the PRONTO in assessing nutritional risk and in predicting malnutrition, as assessed with GLIM criteria. Moreover, we assessed the PRONTO’s usability in newly diagnosed oncology patients under real-world conditions in Spain.

## 2. Materials and Methods

### 2.1. Study Design

This project was a cross-sectional and observational pilot study on the feasibility of a novel nutritional screening tool, PRONTO, in oncology patients. Patients from the medical oncology and radiotherapy services from Hospital La Paz and Hospital Fundación Jiménez Díaz in Madrid, Spain were invited to participate. Fourteen oncologists (Hospital Fundación Jiménez Díaz = 6, Hospital La Paz = 8) were responsible for patient recruitment and participated in usability and NPS ratings.

### 2.2. Healthcare Professionals (HCPs)

At both sites, oncologist teams received didactic lectures on nutrition from endocrinologist physicians of the clinical nutrition team, and practical hands-on learning of proper PRONTO use to reduce time for patients to initial nutrition intervention when nutritional risk was present. Additionally, a training session was conducted via videoconference to ensure consistency across the participating oncologists. The session detailed the project’s objectives and procedures, explained the nature and formulation of the PRONTO questions, and emphasized the importance of consecutive patient recruitment to minimize selection bias. Each oncologist interviewed a minimum of ten patients to gain a basic experience using the PRONTO tool to evaluate its feasibility. After completing the fieldwork, HCPs were asked to complete the SUS electronically. The SUS, proposed by Brooke in 1996, is a widely used questionnaire for assessing the usability of a system [25], which has been extensively used in the health field and was transculturally validated into Spanish in 2020 [24]. The scale scores from 0 (unacceptable) to 100 (acceptable), and values are considered acceptable in terms of usability from 68 points [13,14]. Healthcare professional participants were also asked about their level of recommendation of the PRONTO tool to their department colleagues with the Net Promoter Score (NPS): “On a scale of 0 to 10, how likely are you to recommend the PRONTO questionnaire to a colleague?”. Promoters (scores 9–10), passives (7–8), and detractors (0–6) were defined per standard NPS categories. NPS was computed as 100 × [(promoters/total) − (detractors/total)], yielding a range from −100 to +100.

### 2.3. Patients

The fieldwork was conducted between January 2023 and September 2023. In each site, the first 100 patients of both genders and ≥18 years of age, presenting to medical oncology and radiotherapy oncology services after cancer diagnosis, or with recurrence of cancer with no oncology treatment were included. No prior nutritional evaluation of patients was completed prior to the first oncologist’s visit to avoid bias.

Data collected from each patient included sex, age, type and stage of the oncological process, and the score on the Eastern Cooperative Oncology Group (ECOG) performance scale [26,27]. All patients were screened for nutritional risk with the PRONTO tool and assessed for malnutrition using GLIM criteria, both performed by oncologists. Oncologist therapeutic approach, recommendation for oral nutritional supplement (ONS), and referral to the Hospital Nutrition Service were recorded.

### 2.4. Sample Size

A sample size of 200 patients was determined to be sufficient for assessing the primary study objective as well as to ensure that the medical teams of both hospitals were equally involved in using the PRONTO tool and GLIM criteria on their eligible patients and developed a good level of comfort in using them. It was anticipated that each clinician at the participating hospitals would perform at least 20 nutritional evaluations during the study period, which would give a sample size of 10 clinicians at minimum to complete the SUS and NPS to evaluate acceptability.

### 2.5. Ethical Review

This study protocol was reviewed and approved by the “Comité de Ética de la Investigación con Medicamentos del Hospital Universitario La Paz” and by the “Comité de Ética de la investigación de la Fundación Jímenez Díaz” and written informed consent was obtained from all participants.

### 2.6. Statistical Analyses

Descriptive statistics for continuous (mean ± SD) and categorical [*n* (%)] data were calculated for the relevant participant characteristics (e.g., age, sex, and comorbid conditions). Comparisons of categorical variables were performed using the chi-square test or Fisher’s exact test, as applicable. For continuous variables, the independent samples *t*-test was used for comparisons between two groups, and ANOVA when comparisons involved more than two groups; alternatively, the Mann–Whitney U test was applied depending on data distribution. For longitudinal comparisons, the paired samples *t*-test was used, with each individual’s own value serving as the control. For all comparisons, a two-tailed statistical significance level of 0.05 was considered. The responses of healthcare professionals regarding feasibility were analyzed using descriptive statistics: mean ± standard deviation (SD) and median (interquartile range) for the results of the SUS and NPS scales, and *n* (%) for qualitative responses. All analyses were performed using R (Version 4.3.2).

## 3. Results

### 3.1. PRONTO Feasibility

**Figure 1:**
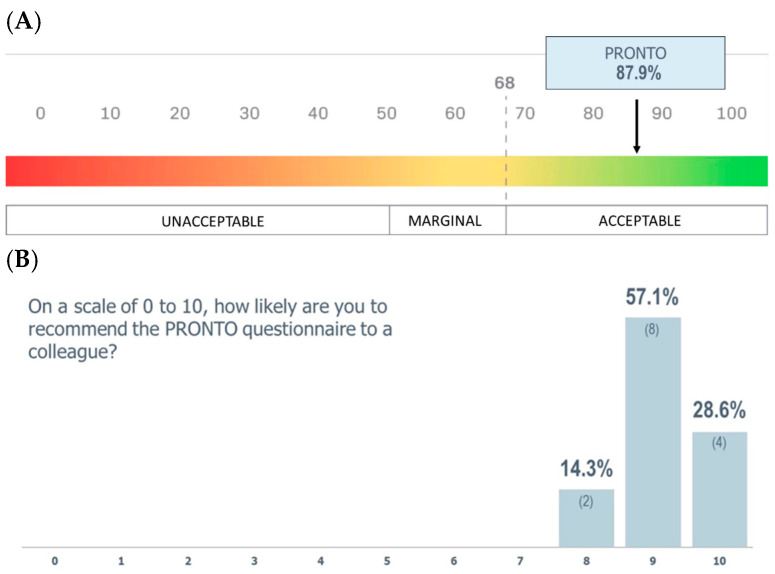
Phase 1 healthcare professional SUS and NPS results. (**A**) System Usability Scale (SUS). Mean score 87.9 ± 7.5 (95% CI: 83.6–92.2), indicating excellent usability (SUS > 80). The SUS is a validated 10-item questionnaire ranging from 0 to 100, where higher scores indicate better usability. (**B**) Net Promoter Score (NPS). Responses of 9–10 were categorized as promoters, 7–8 as passives, and 0–6 as detractors. The value of the index +85.7 was classified as excellent (NPS > 50).

Healthcare professionals (*n* = 14) rated the PRONTO with a mean SUS score of 87.9 ± 7.5 points (95% CI: 83.6–92.2) (excellent, SUS between 80 and 90 points) (Figure 1A). They were also likely to recommend the PRONTO to a colleague, with a mean NPS of 9.1 ± 0.7 points (95% CI: 8.7–9.5) and an index of +85.7 (excellent, NPS > 50) (Figure 1B).

### 3.2. Patient Results

Data from 200 patients presenting to medical oncology and radiotherapy oncology services after cancer diagnosis or with recurrence of cancer with no oncology treatment were collected (Table 1). More than half of patients were women (50.5%) with mean age of 65.3 ± 12.8 years (Table 1). The majority of patients (59.5%, *n* = 119) were fully active via the ECOG performance scale (Table 1).

Lung cancer (34.5%), colon and rectal cancer (22.5%), breast cancer (7.5%), and head and neck cancer (7.0%) were the most frequently represented in the sample (Table 1). Late-stage cancer (stage III or IV) was observed in more than half of our sample (*n* = 138). Lung (84.1%, *n* = 58/69), kidney (71.4%, *n* = 5/7), gastric (66.7%, *n* = 6/9), colon and rectal (64.4%, *n* = 29/45), head and neck (50.0%, *n* = 7/14), and breast (33.3%, *n* = 5/15) cancers had the highest incidences of late-stage cancer (Table 2).

When comparing which cancer types, stages, and ECOG scores affirmatively responded among the domains, there were no statistically significant differences among groups (*p* < 0.999, *p* = 0.921, *p* = 0.585, respectively). A slight trend was identified between the strength loss question and the ECOG stage (*p* = 0.585). Nearly two-thirds of patients (62.4%, 53/85) who declared loss of strength and mobility had an increased ECOG performance status score, compared to those with reduced appetite and food intake (54.8%, 46/84), and body weight loss (52.1%, 50/96) (*p* = 0.585) (Table 3).

More than half (57.0%, *n* = 114) of sampled patients had malnutrition via the GLIM, with the majority being moderate (76.3%, *n* = 87/114) followed by severe (23.7%, *n* = 27/114) malnutrition (Table 4). Among the represented cancers, gastric (100.0%, *n* = 9/9), kidney (85.7%, 6/7), colon and rectal (62.2%, *n* = 28/45), and lung (58.0%, *n* = 40/69) had a high incidence of malnutrition (Table 4). By cancer stage and malnutrition diagnosis, 36.4% (*n* = 8/22) presented malnutrition at stage I, 52.9% at stage II (*n* = 18/34), 46.9% at stage III (*n* = 23/49), and 71.9% (*n* = 64/89) at stage IV. (Table 4).

Nearly two-thirds of all patients (62.0%, *n* = 124/200) answered at least one out of three PRONTO questions affirmatively. Forty-eight percent of all patients (*n* = 96/200) reported involuntarily weight loss (5% to 10% or more) in the last 3–6 months, 42.0% (*n* = 84/200) had been eating less than usual in the last week, and 42.5% (*n* = 85/200) had lost strength or felt weaker than usual since their last consultation. More than a third of patients with malnutrition (42.1%, *n* = 48/114) answered affirmatively for all questions (Table 5). Thus, 78.9% (*n* = 90/114) of patients with malnutrition reported having lost weight, 64.0% (*n* = 73/114) had eaten less than usual, and 64.9% (*n* = 74/114) had lost strength.

Overall, nearly all (sensitivity = 90.4%, *n* = 103/114) of the patients who had an affirmative response to any of the three PRONTO questions had malnutrition via GLIM criteria. More than three-quarters (specificity = 75.6%, *n* = 65/86) of the patients that did not respond affirmatively to any of the PRONTO questions did not have a malnutrition diagnosis. The PRONTO’s positive predictive value (PPV) and negative predictive values (NPVs) were 83.1% (*n* = 103/124) and 85.5% (*n* = 65/76), respectively.

More than half of all patients (57%, *n* = 114/200) had malnutrition, and more than a quarter of patients’ treatment plans (27.2%, *n* = 31/114) did not include the care of a clinical nutrition consultation or the use of an oral nutritional supplement (ONS) (Table 6). This trend was similar even when accounting for malnutrition severity (Table 6).

## 4. Discussion

### 4.1. Malnutrition Is Present Among Oncology Outpatients at Initial Consultation Visits

Our sample indicated that more than half of oncology outpatients at their initial consultation visit had malnutrition. Our prevalence (57%) slightly exceeds PreMiO (51.6%) [6] and NUTRIONCO (39.4%) [28] studies. These differences could be explained by several factors: first, in both the PreMiO and NUTRIONCO studies, nutritional assessment was performed using the Mini Nutritional Assessment^®^ (MNA), while in the present study we used the GLIM criteria; second, types of cancers and patient number are different in the three cohorts; third, data were collected in different periods and in different countries (Italy vs. Spain). Notably, in this study, nearly 6 in 10 oncology outpatients had malnutrition, which may have repercussions on treatment progression and, therefore, cancer progression. This vicious cycle significantly influences the tolerance and acceptance of anti-cancer therapies, affects overall quality of life, diminishes the efficacy of chemotherapy regimens, ultimately impacting the final prognosis [9,29,30,31,32].

Among gastric, kidney, colon and rectal, and lung cancers, malnutrition incidence was high in our sample. Outside of cancer type, when considering cancer staging at diagnosis, more than a third of stage I patients with cancer had malnutrition. This number rose as stage severity increased to about half at stages II and III. Once reaching stage IV, nearly three-quarters of patients experienced malnutrition. This high incidence of malnutrition at stage IV is similar to previous studies [33,34,35,36,37,38,39,40,41]. These incidence percentages should not be assessed in isolation due to the location of the cancer, the small sample size, and that the study was not designed to collect the prevalence of malnutrition in all oncological populations and processes.

While there is awareness that the type and staging of an oncological process can be associated with malnutrition, we were surprised to find these incidences at the first oncology consultation visit. Malnutrition not only accelerates cancer progression, but also significantly influences the tolerance and acceptance of anti-cancer therapies, diminishes the efficacy of chemotherapy regimens, and impacts the final prognosis [9,29,30,31,32]. These incidence rates show the crucial need for multidisciplinary, multi-component nutrition interventions to support patients’ nutrition status early in their cancer journey.

### 4.2. PRONTO Is Simple to Screen for Malnutrition and Streamline Nutrition Care

The PRONTO provides a simple, usable, and feasible way to screen the risk of malnutrition among oncological patients early in their cancer journey. This provides the opportunity for healthcare professionals to intervene with nutritional care such as a referral for a clinical nutrition consultation and/or an ONS when indicated. SUS and NPSs for the PRONTO were high among healthcare professionals, indicating good acceptability for use in clinical practice [24,25]. Our pilot results indicate that the PRONTO has a good sensitivity, specificity, PPV, and NPV among medically complex oncology patients in the outpatient setting when compared to other well-known screening tools such as the MUST, MST, NRS-2002, or Mini Nutritional Assessment-Short Form (MNA-SF) [42].

Among patients that had affirmative PRONTO responses, nutritional risk was present in nearly 9 in 10 patients. The PRONTO utilizes two of the GLIM’s phenotypic criteria (weight loss (body weight loss), reduced muscle mass (strength and mobility)) and one etiologic criteria (reduced food intake or assimilation (appetite and food intake)). These similarities may have contributed to the good concordance seen via sensitivity results. Different combinations of affirmative responses to the PRONTO domains indicated that there may be different presentations of malnutrition dependent on cancer type, location, stage, metastases presence, and ECOG performance status score. The use of the PRONTO tool in a medically complex outpatient oncology population at first consultation is a valuable tool to identify persons with high-risk signs for malnutrition and treat it when indicated. A prospective cohort study that followed hospitalized oncology patients for 12 months after discharge demonstrated that the nutritional risk (NR) identified by the PRONTO (sensitivity of 90.7%) was an independent predictor of 12-month mortality. These findings reinforce that the early identification of nutritional risks with tools like the PRONTO is crucial for favoring early initiation of nutritional interventions that could potentially mitigate adverse outcomes in oncology patients [43].

The PRONTO does not seek to replace existing malnutrition screening or risk assessment tools but rather aims to complement and validate them within the context of daily practice in medical and radiation oncology. Its purpose is to facilitate the early identification of nutritional risk among medically complex patients with cancer. This protocol is designed to be straightforward, evidence-based, and cost-effective, enabling oncologists to recognize and address nutritional risks without disrupting their daily clinical routines or requiring specialized knowledge in nutrition. By integrating the PRONTO, healthcare professionals can enhance their current nutritional assessments, ensuring that patients receive timely and appropriate nutritional interventions alongside their standard cancer care.

### 4.3. Incorporating Nutritional Risk Screening as Part of Nutrition-Focused Quality Improvement Programs

Nutrition-focused quality improvement programs (QIPs) have been found to improve the identification and management of patients at-risk for/or with malnutrition, whilst yielding significant improvements in health and economic outcomes among different patient populations (e.g., medical, surgical, cardiovascular, and diabetes, etc.), especially oncology patient populations [44,45,46,47,48,49]. QIPs are used to improve care systematically by standardizing processes and care structures; the goal is to reduce variation and inform optimized nutrition care processes that are sustainable over time and improve outcomes for patients and healthcare systems. Nutrition-focused QIPs have focused on simple steps including malnutrition screening, nutrition education, and counseling among patients identified as at-risk for/or with malnutrition, prompt initiation of a disease-specific ONS, re-education at discharge, and follow up [44,45,46,47,48,49]. Secondary analysis of a large nutrition-focused QIP conducted in the United States looking at patients with cancer at-risk for/or with malnutrition in a hospital-based, comprehensive study documented significant reductions in 30-day readmission rates and length of stay, resulting in potential cost savings of over USD 3800 per patient [44]. Similar improved outcomes have been observed in outpatient cohorts of patients with cancer participating in nutrition-focused QIPs implemented in outpatient clinics offering optimized nutrition care to patients presenting for care either for continuous disease management or post-hospitalization [46,48,49].

In this study, although nearly three-quarters of patients received a nutrition intervention post-identification of nutritional risk or status, more than a quarter of patients did not; therefore, highlighting the importance of comprehensive, nutrition-focused QIPs to ensure the consistent and continuous use of the PRONTO, which will in turn inform next steps as in nutrition education and counseling, ONS intervention when needed, and follow up with the intent to improve patient outcomes and reduce healthcare costs. Involvement of oncologists in nutritional risk screening through the PRONTO in outpatient clinics where the presence of nutrition team staff is scarce is imperative in ensuring that no oncology patient remains unidentified, undiagnosed, and undertreated to optimize their cancer care treatment and improve their outcomes.

### 4.4. Strengths

This study highlighted that the PRONTO tool was feasible in screening the risk of malnutrition among oncology patients’ first visit with good sensitivity, good PPV, and good NPV. Clinicians did not share significant barriers or challenges they encountered during their use of the PRONTO, and the simplicity of the tool facilitated its integration into routine clinical practice. This was highlighted with high acceptability and promotion scores of the PRONTO tool recorded by all healthcare professionals participating in the SUS (close to 90 points) and excellent NPS (+85.7).

### 4.5. Limitations

This study has several limitations inherent to all non-randomized clinical and pilot studies. This pilot study utilized a non-statistically powered sample size; therefore, its results cannot be extrapolated to all patients with cancer or to all oncologists. The PRONTO was also not compared to other nutrition screening tools as this study focused on feasibility, which would be necessary to establish differences or similarities. However, de Miranda [43] recently reported that the PRONTO performs similarly to other nutrition screening tools (e.g., PG-SGA and NRS-2002) with a reported sensitivity rate of 90.7% and 85% PPV among patients with cancer assessed 48 h post-hospitalization. Only basic patient demographic information from a sample of 200 was collected, so we were not able to assess nutritional risk trends among specific patient groups or establish prevalence, i.e., those with different comorbidities, diagnoses, or other socio-economic backgrounds. Patients were not followed over time, which limited the ability to assess nutrition improvements over time, especially post-intervention improvements. Without such fine-tuning, the study results are not generalizable to the entire oncology population; however, this study employed a sample representing different types of patients with cancer with insights from differing specialized oncologists. The PRONTO protocol, originally designed as a nutritional awareness tool for patients with cancer, demonstrated its feasibility in daily clinical practice through this study. The findings suggest that the PRONTO may have potential applications beyond awareness, serving as a screening tool for nutritional risk, similar to established tools like the MUST or MST, or as a screening tool for muscle mass and/or function loss. These additional functionalities could make the PRONTO a valuable addition to oncology care, allowing healthcare professionals to efficiently identify and address nutritional risks. However, further research is necessary to validate its use as a screening method and to compare its predictive capabilities against other validated tools in larger, more diverse outpatient oncology populations, whilst taking note of comorbidities and other patients (e.g., cancer type, tumor site, and tumor stage, etc.). Additionally, further research should study different oncologist subspecialties for further details on the acceptability, feasibility, and scalability of the PRONTO across different settings of care (e.g., hospital and outpatient clinics, etc.).

Finally, future research is needed to examine how nutrition-focused QIPs that include nutrition screening with the PRONTO, education, and nutrition intervention including ONS, can lead to better patient outcomes including improved response to oncology therapy and quality of life, reduction in healthcare resource use, as well as improved clinical and nutritional decision making, and improved clinical work flow.

## 5. Conclusions

To our knowledge, this is the first study evaluating the feasibility of the PRONTO nutritional screening tool in outpatient clinical practice. The PRONTO is a feasible tool for nutritional risk screening in oncology patients, with good concordance with the GLIM. With the high incidence of malnutrition observed at the first oncology consultation visit, there is a critical need for future studies to identify adults who are at the greatest risk of malnutrition. Specifically, studies should focus on pinpointing those most likely to benefit from targeted and comprehensive nutrition interventions, aiming to improve overall health and cancer outcomes.

## Figures and Tables

**Table 1:** Patient demographics, *n* = 200.

Age, Mean ± SD	65.3 ± 12.8
**Sex**, % (*n*)	
Male	49.5 (99/200)
Female	50.5 (101/200)
**Cancer Type**, % (*n*)	
Lung	34.5 (69/200)
Colon and Rectal	22.5 (45/200)
Breast	7.5 (15/200)
Head and Neck	7.0 (14/200)
Gastric	4.5 (9/200)
Bladder	4.0 (8/200)
Kidney	3.5 (7/200)
Other Cancers	16.5 (33/200)
**Cancer Stage**, % (*n*)	
0	3.0 (6/200)
I	11.0 (22/200)
II	17.0 (34/200)
III	24.5 (49/200)
IV	44.5 (89/200)
**ECOG Performance Status**, % (*n*)	
0	59.5 (119/200)
1	30.0 (60/200)
2	6.5 (13/200)
3	3.5 (7/200)
4	0.5 (1/200)

ECOG = Eastern Cooperative Oncology Group [25,26]. Other cancer types included esophageal, sarcomas, endometrium-uterine, liver, ovarian, testicle, penis, skin, and pancreatic cancers.

**Table 2:** Patient cancer types by stage at the time of the first oncology consultation (*n* = 200), % (*n*).

	Lung	Colon and Rectum	Breast	Head and Neck	Gastric	Bladder	kidney	Others
**Cancer Stage ** ***n *** ** = 200**								
0 *n* = 6	0 (0/69)	2.2 (1/45)	20 (3/15)	7.1 (1/14)	11.1 (1/9)	0 (0/8)	0 (0/4)	0 (0/33)
I *n* = 22	8.7 (6/69)	4.4 (2/45)	33.3 (5/15)	14.3 (2/14)	0 (0/9)	0 (0/8)	14.3 (1/4)	18.2 (6/33)
II *n* = 34	7.2 (5/69)	28.9 (13/45)	13.3 (2/15)	28.6 (4/14)	22.2 (2/9)	37.5 (3/8)	14.3 (1/4)	12.1 (4/33)
III *n* = 49	15.9 (11/69)	42.2 (19/45)	13.3 (2/15)	28.6 (4/14)	44.4 (4/9)	25 (2/8)	14.3 (1/4)	18.2 (6/33)
IV *n* = 89	68.1 (47/69)	22.2 (10/45)	20 (3/15)	21.4 (3/14)	22.2 (2/9)	37.5 (3/8)	57.1 (4/4)	51.5 (17/33)

Note: Other cancer types included esophageal, sarcomas, endometrium-uterine, liver, ovarian, testicle, penis, skin, and pancreatic cancers.

**Table 3:** Affirmative answers to PRONTO questions by cancer type, stage, and ECOG performance status score, % (*n*).

	1 Body Weight Loss	2 Appetite and Food Intake	3 Strength and Mobility
Overall *n* = 200	48.0 (96/200)	42.0 (84/200)	42.5 (85/200)
Cancer Type *n* = 200			
Lung *n* = 69	32.3 (31/96)	35.7 (30/84)	38.8 (33/85)
Colon and Rectal *n* = 45	27.1 (26/96)	19.0 (16/84)	17.6 (15/85)
Breast *n* = 15	3.1 (3/96)	1.2 (1/84)	3.5 (3/85)
Head and Neck *n* = 14	5.2 (5/96)	8.3 (7/84)	4.7 (4/85)
Gastric *n* = 9	9.4 (9/96)	9.5 (8/84)	7.1 (6/85)
Bladder *n* = 8	2.1 (2/96)	3.6 (3/84)	3.5 (3/85)
Kidney *n* = 7	5.2 (5/96)	4.8 (4/84)	4.7 (4/85)
Other Cancers *n* = 33	15.6 (15/96)	17.9 (15/84)	20.0 (17/85)
Cancer Stage *n* = 200			
0 *n* = 6	1.0 (1/96)	1.2 (1/84)	0.0 (0/85)
I *n* = 22	8.3 (8/96)	8.3 (7/84)	8.2 (7/85)
II *n* = 34	18.8 (18/96)	19.0 (16/84)	14.1 (12/85)
III *n* = 49	19.8 (19/96)	17.9 (15/84)	15.3 (13/85)
IV *n* = 89	52.1 (50/96)	53.6 (45/84)	62.4 (53/85)
ECOG Performance Status *n* = 200			
0 *n* = 119	52.1 (50/96)	45.2 (38/84)	37.6 (32/85)
1 *n* = 60	36.5 (35/96)	39.3 (33/84)	43.5 (37/85)
2 *n* = 13	7.3 (7/96)	9.5 (8/84)	12.9 (11/85)
3 *n* = 7	4.2 (4/96)	4.8 (4/84)	5.9 (5/85)
4 *n* = 1	0.0 (0/96)	1.2 (1/84)	0.0 (0/85)

PRONTO = PROtocol for NuTritional risk in Oncology. ECOG = Eastern Cooperative Oncology Group [25,26]. Note: Other cancer types included esophageal, sarcomas, endometrium-uterine, liver, ovarian, testicle, penis, skin, and pancreatic cancers.

**Table 4:** Prevalence and GLIM severity by cancer type, cancer stage, and ECOG performance status, % (*n*).

	Malnutrition	Moderate Malnutrition	Severe Malnutrition
Overall *n* = 200	57.0 (114/200)	76.3 (87/114)	23.7 (27/114)
Cancer Type *n* = 200			
Lung *n* = 69	58.0 (40/69)	72.5 (29/40)	27.5 (11/40)
Colon and Rectal *n* = 45	62.2. (28/45)	89.3 (25/28)	10.7 (3/28)
Breast *n* = 15	26.7 (4/15)	100.0 (4/4)	0.0 (0/4)
Head and Neck *n* = 14	42.9 (6/14)	83.3 (5/6)	16.7 (1/6)
Gastric *n* = 9	100.0 (9/9)	77.8 (7/9)	22.2 (2/9)
Bladder *n* = 8	50 (4/8)	75.0 (3/4)	25.0 (1/4)
Kidney *n* = 7	85.7 (6/7)	100.0 (6/6)	0 (0/6)
Other Cancers *n* = 33	51.5 (17/33)	47.1 (8/17)	52.9 (9/17)
Cancer Stage *n* = 200			
0 *n* = 6	16.7 (1/6)	100.0 (1/1)	0.0 (0/1)
I *n* = 22	36.4 (8/22)	75.0 (6/8)	25.0 (2/8)
II *n* = 34	52.9 (18/34)	77.8 (14/18)	22.2 (4/18)
III *n* = 49	46.9 (23/49)	95.7 (22/23)	4.3 (1/23)
IV *n* = 89	71.9 (64/89)	68.8 (44/64)	31.3 (20/64)
ECOG Performance Status *n* = 200			
0 *n* = 119	47.1 (56/119)	82.1 (46/56)	17.9 (10/56)
1 *n* = 60	70.0 (42/60)	73.8 (31/42)	26.2 (11/42)
2 *n* = 13	76.9 (10/13)	60.0 (6/10)	40.0 (4/10)
3 *n* = 7	71.4 (5/7)	60.0 (3/5)	40.0 (2/5)
4 *n* = 1	100.0 (1/1)	100.0 (1)	0.0 (0/1)

ECOG = Eastern Cooperative Oncology Group [25,26]. GLIM = Global Leadership Initiative on Malnutrition. PRONTO = PROtocol for NuTritional risk in Oncology. Note: Malnutrition defined by diagnosis via the GLIM. Note: Other cancer types included esophageal, sarcomas, endometrium-uterine, liver, ovarian, testicle, penis, skin, and pancreatic cancers.

**Table 5:** Variability of GLIM diagnoses by affirmative PRONTO screening tool responses, % (*n*).

		Body Weight Loss (BWL)	Appetite and Food Intake (AFI)	Strength and Mobility (SM)	GLIM Diagnosis, % (*n*)	No GLIM Diagnosis, % (*n*)
All 3	24.5 (49/200)	YES	YES	YES	42.1 (48/114)	1.2 (1/86)
BWL + AFI	9.5 (19/200)	YES	YES	NO	16.7 (19/114)	0.0 (0/86)
BWL + SM	7.5 (15/200)	YES	NO	YES	13.2 (15/114)	0.0 (0/86)
BWL Only	6.5 (13/200)	YES	NO	NO	7.0 (8/114)	5.8 (5/86)
AFI Only	3.5 (7/200)	NO	YES	NO	1.8 (2/114)	5.8 (5/86)
SM Only	6.0 (12/200	NO	NO	YES	6.1 (7/114)	5.8 (5/86)
AFI + SM	4.5 (9/200)	NO	YES	YES	3.5 (4/114)	5.8 (5/86)
None	38.0 (76/200)	NO	NO	NO	9.6 (11/114)	75.6 (65/86)
Totals	100.0 (200/200)	48.0 (96/200)	42.0 (84/200)	42.5 (85/200)	57.0 (114/200)	43.0 (86/200)

GLIM = Global Leadership Initiative on Malnutrition.

**Table 6:** Nutrition treatment of patients with malnutrition identified via GLIM, % (*n*).

	All Malnutrition *n* = 114	Moderate Malnutrition *n* = 87	Severe Malnutrition *n* = 27
Clinical Nutrition Consultation Only	2.6 (3/114)	2.3 (2/87)	3.7 (1/27)
ONS Only	51.8 (59/114)	55.2 (48/87)	40.7 (11/27)
Clinical Nutrition Consultation + ONS	18.4 (21/114)	14.9 (13/87)	29.6 (8/27)
No Nutritional Intervention	27.2 (31/114)	27.6 (24/87)	25.9 (7/27)

ONS = oral nutritional supplement. Note: Malnutrition defined by diagnosis via Global Leadership initiative on Malnutrition (GLIM).

## Data Availability

Data supporting reported results can be found by directly contacting the correspondence authors through german.guzman1@abbott.com and jorge6de@gmail.com.
